# Medication use review: does it have a future and potential in Latvia? The opinion of pharmacists after the pilot project

**DOI:** 10.1186/s40545-023-00551-2

**Published:** 2023-03-27

**Authors:** Madara Paidere, Jūlija Pavlovska, Ieva Rutkovska, Dace Ķikute, Ieva Salmane-Kuļikovska

**Affiliations:** 1 Department of Applied Pharmacy, Riga Stradins University, Riga, Latvia; 2 Pharmacists’ Society of Latvia, Riga, Latvia

**Keywords:** MUR, Medication review, Barriers, Pharmacist, Community pharmacy

## Abstract

**Background:**

Given that the Medication Use Review (MUR) can be used as a tool to improve the quality, safety, and appropriate use of medications, it has been implemented in several countries around the world. The MUR pilot project was carried out in Latvia, followed by this study to identify obstacles, favorable factors, and benefits of this service from the perspective of pharmacists, being one of the key stakeholders.

**Methods:**

Qualitative data were obtained through a semi-structured focus group interview with pharmacists participating in the project. Inductive thematic analysis was performed on transcript to describe potential gains, obstacles, and prerequisites for implementing the MUR service from the pharmacist’s perspective.

**Results:**

Lack of payment, cooperation with physicians, problems with patient involvement and insufficiency of competence, were identified as the main barriers to implementation of the MUR service as a pharmacist-led service in community pharmacy. However, there were also contributing factors for MUR service, such as the interest of patients and pharmacists involved in the project, the support from the employer, the benefit for physicians, the improvement of patient health literacy and medication adherence.

**Conclusions:**

Despite the potential obstacles, the findings in Latvia, as well as other countries, show that the MUR service contributes to the benefit of patients, pharmacists and also physicians; therefore, further steps should be taken to eliminate obstacles and gain additional insights to implement the MUR service in Latvia.

## Background

The Medication Use Review (MUR) is widely available worldwide as a daily pharmacy service. In some countries, such as UK [1], USA [2] and Australia [3], it is a part of patient-centered care and is reimbursed by the state.

According to legislation in Latvia, the main duty of a pharmacist is to provide appropriate pharmaceutical care; however, the regulations of the Cabinet of Ministers of the Republic of Latvia (Regulation No. 288 “Regulations Regarding Operating of Pharmacies”) regulating the operation of pharmacies do not include any specific services that would promote the development of patient-centered care. These regulations stipulate that the pharmacist must provide pharmaceutical care, in which blood pressure control and cholesterol assessment are included as an additional service, but in legislation MUR is not included as a service that can be provided by a pharmacist.

The problem of polypharmacy increases globally as well as in Latvia, where the number of patients simultaneously using five and more medications rises, contributing to an increasing number of medication-related problems [4]. The MUR pilot project in Latvia was organized in the period from March 2019 to October 2019. It was part of the MUR pilot project in 11 Eastern European countries (Estonia, Latvia, Lithuania, Poland, Croatia, Bosnia and Herzegovina, Hungary, Romania, Bulgaria, Slovakia), and Iran. The aim of this project was to acknowledge opportunities to advance the MUR service [5].

## Methods

### Aim

To identify pharmacists’ views on the results of the pilot project, the necessity, readiness, and obstacles to the implementation of MUR service in Latvia: potential gains, obstacles, and preconditions.

### Design

This was a qualitative, exploratory study using a focus group interview with pharmacists providing a pilot study of the MUR service, in community pharmacies in Latvia. Considering previous studies and focus group interview guides [6, 7], the interview guidelines were developed. The open-ended interview questions covered topics such as the organization of the pilot project (quality of preparatory work, technical support, etc.), outcome of the pilot project, opinion about the necessity of the MUR service project in Latvia, readiness and qualification of pharmacists, potential threats and opportunities of the project from the pharmacists’ perspective.

### Participant recruitment and characteristics

The five pharmacists who participated in the MUR pilot project in Latvia from March 2019 to October 2019 participated in one focus group interview. Five pharmacists who had a master’s degree in clinical pharmacy and were working in five different community pharmacies participated in the pilot project. Pharmacists underwent a methodology-based training program by the University of Tartu prior to the pilot project. The MUR service was offered to patients identified and approached by a pharmacist or general practitioner. A Medication card was created during the MUR service for patients, which schematically indicated the medication used, the dose, time, route and frequency of administration. The criteria for the inclusion of patients were as follows: patients with polypharmacy (taking at least five medications); at least 18 years; it was possible to accurately identify the indications and medications used; have signed the informed consent form.

### Data collection

Data were collected from a one semi-structured focus group interview, held at ZOOM on December 9, 2020, led by two researchers. The interview was recorded and subsequently transcribed verbatim. The interview was anonymized to remove all identifying information and to protect the confidentiality of pharmacists.

### Data analysis

Inductive thematic analysis as described by Braun and Clarke [8] was performed on transcript to describe potential gains, obstacles, and prerequisites for implementing the MUR service from the pharmacist’s perspective.

Based on the assumptions of Braun and Clarke, six consecutive phases were used to obtain results: familiarization with the data, generating initial codes, searching for themes, reviewing themes, defining and naming themes, and producing the report [8].

The statements were coded, compared for similarities and differences, and then categorized using MAXQDA software. To establish the reliability of the coding process, two researchers independently performed the coding procedure; afterwards, the data were merged. Themes and categories are displayed in Table 1.

**Table 1:** Identified themes and categories

Obstacles to MUR project implementation	Funding
	Lack of pharmacists with adequate competence
	Systemic factors
	Publicity
	Involvement of doctors and patients
	Employer’s insufficient support
Favorable factors for the MUR project	Employer’s support
	The interest of the pharmacists involved in pilot project
	The interest of the patients involved in pilot project
Benefits of the MUR project	Time saving for physicians
	Prestige of the profession of pharmacist
	Improvement in health literacy and adherence
	Promoting the rational use of medicines

## Results

The themes and categories resulting from the data analysis are discussed below:

### Obstacles to MUR project implementation

The pilot project identified a number of obstacles that hinder and need to be addressed to introduce MUR as a service in Latvia.

#### Funding

In this pilot project, pharmacists were not paid for providing the MUR service. It required additional time and knowledge, so the interviewees believed that additional payment is necessary to provide this service. *“I think it is extra work, … Given that this project required more effort, more preparation, I think yes—any work has to be paid for.”* (P2) Patients who participated in this project often wanted to make sure that this service was free for them. There are groups of the population that could be particularly vulnerable. *“I doubt the seniors will have the money for such a service.”* (P1) Most focus group participants believed that a potential solution could be the reimbursement from the NHS service.

#### Lack of pharmacists with adequate competence

Currently, there are no pharmacy qualification criteria for participating in the MUR project. According to pharmacists, this service would require either a clinical pharmacist’s degree and/or training to be able to ensure a quality MUR service. *“Our consultations were advanced; we were looking for specific medication-related problems.”* (P1) One way would be to check the pharmacist’s competency before providing the service. Another way would be to include providing the necessary skills through continuing education. If this service could be organized more widely, the shortage of pharmacists could be a problem. This service must be provided without disrupting the work of the pharmacy, so additional staff would be needed.

#### Systemic factors

The information currently used in the service on the patient’s medical history, used medications, and current diseases was obtained only from patients. Consequently, there is a high risk of a lack of information or possibility that information is incorrect, disallowing appropriate consultation with complete information on the current condition and all medications used. *“The obstacle is the availability of patient information—the patient only showed what he wanted to show when came to the consultation.”* (P3). Pharmacists noted that the lack of appropriate information about the medications used by patients is one of the challenges. There is currently no source containing all medical data about the patient. Such system could help to divert MUR service to those patients who need it most. “*The best option would be to build a one system for all health care professionals. If a pharmacist is aware that a patient has medication-related problems, they can be referred immediately to the MUR service. By advertising this service nationally, people will have more information about it and will be more open to such an opportunity.* (P1).

It was identified in the interviews that the main challenges were caused by the data entry system (RedCAP). There were problems with the data entry process, and it was difficult to use it during the consultations. Therefore, additional time was required to use the program to enter all patient’s related data in the program. Consequently, the project participants admitted that the data collection process caused them problems.

According to the opinion expressed by pharmacists, to respect confidentiality, a separate room is required to ensure the service; it cannot be provided in the consumer service area of the pharmacy. A segregated room might be an obstacle to a wider deployment of the service. *“To provide the service, I had to agree separately on a room where I could arrange it at a certain time, because there were no such rooms in the pharmacy.”* (P2).

Given that advanced consultation was also provided for patients speaking in the Russian language as well, some pharmacists acknowledged that the language barrier was very cumbersome for pharmacists to provide complete information.

#### Publicity

Pharmacists admitted that promotion is necessary to ensure recognition of this service—promotion can be provided through radio, television or social networks, but information about this service should also be discussed in professional associations of general practitioners. The participation of other healthcare professionals would ensure that the service is more reliable for patients. It would be crucial to demonstrate the benefits of the project, illustrated, e.g., by sample studies and reviews of patients who had participated in the pilot study.

#### Involvement of physicians and patients

Successful service development requires tripartite cooperation between the pharmacist, the physician, and the patient. Interviewed pharmacists noted that initially, patients were confused about the MUR service, especially at the first visit. This was especially the case for patients who were offered to participate in an MUR project in a pharmacy. The patients did not understand the importance of the service, they felt insecure about the consultation*. “Other times there was a situation where the patients did not completely trust you.”* (P1) Lack of trust in the pharmacist as a knowledgeable professional could be identified as an obstacle in the part of patients. In addition, the lack of understanding of the service itself and the expected results of patients was an important aspect.

The pilot project organization determined that the physicians received direct emails and visits from the pharmacists who invited them to participate in the project. Pharmacists admitted that face-to-face visits were more effective because they also increased physicians’ awareness of the service. In direct communication, they evaluated the possibility of the service, acknowledging that there were patients who would need MUR. Despite this, cooperation with physicians should be improved. This could be caused by a lack of understanding of the process and does not understand the added value that the MUR service can provide*. “The main obstacle was the disinterest of physicians. Most of them also did not understand the need for the service and what kind of service a pharmacist can provide.”* (P3).

Furthermore, no doctor feedback was received on the project or on provided consultations.

#### Employer’s insufficient support

The pharmacists involved in the pilot project did not have an exact time during their working hours when they could perform the MUR consultation. Consequently, it was difficult for pharmacists to combine these consultations with the daily duties of pharmacists. *‘In my case, the activities related to this project were not intended to be done during working hours’ (P2).*

Due to these aspects, pharmacists in some cases had to conduct consultations or prepare for them after their working hours. *‘It was hard for me to combine this project with my job, so I usually did it after working hours’ (P1)*. This created an additional burden for pharmacists to participate in the pilot project and be able to provide appropriate consultations.

### Favorable factors for the MUR project

Several aspects encountered by pharmacists within the pilot project indicate that such a service would be useful and necessary or could contribute to its implementation and development in Latvia.

#### Employer’s support

The support of the employer was stated to be an important factor. Pharmacists admitted that it was challenging to combine MUR consultation with daily work. The attitude of the employer was different in each case. It was important to adjust the consultation time to avoid disruption of the organization of work in the pharmacy. Therefore, it was important to agree on the consultation time with the patient and to find time to prepare for it. “*It is not necessary to prepare especially for the first visit, because everything is already available, but it is mandatory to prepare for the next consultations. Some time is necessary, a day, to find the unclear issues or those that need additional information.”* (P3).

#### The interest of pharmacists involved in the pilot project

The pharmacists involved in the pilot project were very interested in providing the service, because they felt it raised the prestige of the profession and the desire to help improve patient health literacy and medication adherence that pharmacists are sometimes unable to provide due to their busy schedule. *“[..] people often do not understand why the specific medicine should be used and how to use it properly, and I often do not have the time in the pharmacy to put it all on the shelves and explain it to them properly.”* (P2).

#### The interest of the patients involved in the pilot project

During the project, pharmacists have identified the need for this service from patients. *“People often do not know how to properly take medication and why it is necessary. On a daily basis, professionals often do not have time to explain. When patients receive the information they need, they are very grateful. In my opinion, it seems that there would be a very high demand for such a service.”* (P3) As long as this unmet need exists due to the lack of patient knowledge about the medications used, MUR service will be required. This service can be used by physicians as an aid to compensate for the lack of time, at the same time providing patients with the necessary information by using MUR service.

### Benefits of the MUR project

#### Time saving for physicians

Pharmacists indicated that all stakeholders might benefit from the MUR product. Benefits to physicians include additional information on the patient’s use of medications, as well as considerable time savings. This information could help achieve therapeutic goals. Due to the lack of time for physicians, patients often have limited access to information on the appropriate use of drugs. This responsibility can be delegated to the pharmacist as part of the MUR service, saving time for the physician.

#### Prestige of pharmacists’ profession

The product has the potential to promote the pharmacist’s role in a healthcare team. *“[…] the benefit would be the possibility to promote the pharmacists’ profession—the patient no longer looks at you as a salesperson, but as a medical professional who can help you.”* (P3) Pharmacists involved in the project acknowledged that there was great emotional satisfaction after improving a patient’s therapy as a result of receiving the service. Interviewed pharmacists also indicated that the provision of this service would provide an increased professional self-esteem in each professional individually.

#### Improvement in health literacy and adherence

Interviews revealed that in fact, the greatest benefits are for patients. The development of health literacy and medication adherence can be highlighted as most important. The service helps the patient streamline therapy, evaluate the indication of used OTC products and dietary supplements and possible interactions. Patients’ complaints were adequately addressed and their current problems were solved during the consultation or within the next consultations after consultation with the patient’s physician. An added benefit is the improved relationship with the pharmacist.

#### Promoting the rational use of medicines

Pharmacists emphasized that the patients greatly appreciated the medication list that was prepared for them during the consultation. Patients found it difficult to explain to the pharmacist why they use exact medicine and other necessary information associated with medication use. As a result, patients have received material that shows everything they use on a daily basis. Given that patients have acquired additional knowledge in the use of drugs and practical help, this service is considered to promote rational use of medications.

## Discussion

### Statement of key findings

The main identified beneficiaries of the MUR service for patients are improved knowledge on health associated questions and obtained additional information on appropriate use of medications. Patients have shown high satisfaction after receiving the service [9]. Improvement in health literacy after obtaining this service has also been observed in other studies already carried out in countries, where it is introduced as a daily service in pharmacies, for example, in Thailand an UK [10–12]. The interviewed pharmacists noted that there were different experiences during the consultation depending on the patient’s previous experience and their knowledge of their diagnosis.

One of the motivating factors for pharmacists to participate in this project was to raise the prestige of the pharmacist profession. Other studies also have shown that patients are cautious about the services offered by the pharmacy and that their expectations regarding the services provided by the pharmacist are relatively low [13, 14]. The underestimation of pharmacist knowledge currently prevails not only in the views of the pharmacists interviewed in the pilot project, but also in other countries, where as a result of MUR consultations, patients are introduced to the real benefit that a pharmacist can provide [15, 16]. In this case, the role of the pharmacist in improving public health by achieving the objectives of the MUR can also be highlighted. Ensuring that society is better informed about the MUR project would also increase the awareness of patients about it; it would increase knowledge of possible benefits that patients can receive from the service. The lack of clarity about the service idea, process, and expected results of this service is confusing to both physicians and patients [17]. One of the key identified obstacles during this study was the participation of patients and physicians in the pilot project. Especially, cumbersome was collaboration between pharmacists and physicians. This was identified as one of the most common barriers to the implementation and development of the MUR service in other studies as well [17, 18].

Other studies have shown that physicians are satisfied with the fact that pharmacists provide patients with information on the correct and safe use of drugs [19].

Despite the benefits that the service can bring to both the doctor and the outcome of treatment as a whole, collaboration with physicians was one of the main obstacles identified by the interviewed project participants. Given that without good collaboration skills between the doctor and the pharmacist, the effect of MUR could not reach the maximum effect [20–22]. The possibility of choosing a better way of providing information to doctors through verbal communication would be an opportunity to improve cooperation, providing the doctor with information about the service received by the patient in a short period of time. Our study as well as others have shown that written information to physicians does not ensure the desired proportion of service [21]; therefore, it would be necessary to use other types of promotion. Our study indicated that face-to-face communication with physicians, where pharmacists provide necessary information about the service and potential benefits for doctor and patient, might be more effective, however, this would be time consuming, and a different promotion form is needed.

Practical limiting factors that can influence the wider development of the MUR service were also identified. Interviewed pharmacists stressed that they spend most of their working time dispensing medications, which is also indicated by other studies [23, 24]; therefore, pharmacists need to find additional time to provide MUR service and to coordinate consultation time with the patient. Consequently, the employer must ensure the continuity of pharmacy work, and there might be a need for additional employees [4, 18]. If the pharmacy does not have additional staff to ensure that the pharmacist can provide MUR service without interruptions, the pharmacist’s workload will not promote further service development [25, 26].

An additional obstacle identified in our study was the availability of a separate room for private consultation. This aspect has been identified as an obstacle in several countries, Dubai, UK [15, 27]. This could be a very important aspect, since in several studies, patients have found that privacy is very important for them when choosing to receive such a service [13, 25, 28]. All these organizational factors were also identified in this study.

Interviews indicated that availability of medicinal information about patients was a necessity to the pharmacists. MUR reports have also identified that the quality of recommendations provided by a pharmacist is directly influenced by availability of the information to the service provider [21].

Another aspect that is important to highlight is the competence of the pharmacist who provides the consultation. Other studies also acknowledge that pharmacist competence affects variations in counseling skills. There was a suggestion to include some training for pharmacists in the continuing education process. At the moment, in Latvia, pharmacists have been trained to provide the MUR service only in one university; therefore, such a proposal would be appropriate to increase the knowledge and skills of pharmacists to provide such a service. Other studies have indicated that additional training could provide pharmacists with additional motivation and the ability to provide additional services in the pharmacy [14].

Interviewed pharmacists mentioned that it is necessary to ensure the promotion of this service to raise awareness of MUR in society. As in other studies, lack of awareness is one of the main reasons for the limited use of this service, even in countries where it is already implemented. Experience of other countries indicated that promotional materials such as posters and banners in pharmacies where this service is provided, complimentary paper bags, flyers in places accessible to society are beneficial to promote the service to society [10].

### Strengths and weaknesses

To the authors’ knowledge, this is the first study to examine the outcomes of the MUR pilot project in Latvia, as well as identifies obstacles, favorable factors, and benefits of the service from the pharmacists’ perspective. There are limitations of this study: (1) the five participants in the pilot study were located in the capital city of Latvia and its surrounding; views of the participants in the other regions could provide additional insights and (2) this study focuses on pharmacists’ perspective; opinions of other stakeholders (physicians, institutions, etc.) should be assessed to identify obstacles, favorable factors, and benefits from other perspectives, to better ensure implementation of MUR service in current practice.

### Interpretation

Until now, the development of the pharmacist profession in Latvia has been greatly influenced by historical aspects. Recently, a partial transformation of the profession has taken place, modifying it into patient-centered care rather than product-oriented care. Admittedly, this transformation is happening more theoretically than practically. Several organizational and systemic barriers need to be addressed and overcome before practical implementation.

A similar scenario exists in the interaction between a doctor and a pharmacist. Communication between the doctor and the pharmacist takes place at different levels. Therefore, pharmacists generally do not dare discuss questions related to medications with physicians. This aspect is also discussed in other studies that describe day-to-day communication between these two parties [29, 30].

### Further research

The next step that would facilitate the implementation of the MUR service would be the participation of other stakeholders, to understand the main obstacles and preconditions from their perspective. This is why it would be necessary in the future to conduct a study on the opinion of physicians, patients, and stakeholders such as legislators, employers, and educational institutions for the practical implementation of such a service in Latvia.

## Conclusions

The pharmacists’ perspective and the data of other studies suggest that implementation of the MUR service may bring considerable benefits: (1) improvement of the health literacy of patients and consecutively promotion of rational use of medicines; (2) more significant role of the pharmacist in a patient’s healthcare team; and (3) time saving for the physicians. The pilot project showed that the service was positively viewed by pharmacists and patients; both claimed to have gained knowledge, as well as a better understanding of the patient’s situation and the next steps in the medication use regimen. The experience of other countries shows positive views of physicians about the MUR service. To mitigate obstacles identified by this study, it is essential to: (1) consider ways of facilitating communication between physicians and pharmacists to ensure greater interest from physicians. This could be achieved by raising awareness of MUR in healthcare societies among all stakeholders and the society; (2) implementation of the MUR service requires additional training of the pharmacists; therefore mastering the necessary skills should be a part of the educational programs; (3) regularly updated information regarding patient’s medications and health condition should be accessible for both—a pharmacist and a physician of the patient, e.g., medication use history, hospitalization data, etc.; and (4) improvement of the environment of the pharmacists rendering the MUR service, e.g., separate room for consultations, adequate amount of time both for consulting the patient and preparation for consultations, remuneration, etc. Successful implementation of the MUR service requires the participation of other stakeholders, such as the Ministry of Health, higher education institutions, and professional associations.

The professional association of pharmacists is of particular importance, which would also ensure the organization of further training for pharmacists and which, within the framework of its congresses, have set the goal of reimbursing this service from the state.

## Data Availability

The data sets analyzed during the current study are not publicly available but are available in Latvian from the corresponding author on reasonable request**.**

## Abbreviations

MUR: Medicines use review
OTC: Over-the-counter
